# Long-Term Stability of Nanobubbles Generated via Pressure Oscillation-Hydrodynamic Cavitation: A Rapid Assessment by UV–Vis Spectrophotometry

**DOI:** 10.3390/nano15211613

**Published:** 2025-10-23

**Authors:** Lei Huang, Jiaqi Dong, Ming Chen, Lei Li, Ruichao Zhang

**Affiliations:** 1 College of Intelligent Manufacturing and Control Engineering, Shandong Institute of Petroleum and Chemical Technology, Dongying 257061, China; 2015016@sdipct.edu.cn (L.H.); 2016003@sdipct.edu.cn (L.L.); 2 PipeChina Engineering Technology Innovation Co., Ltd., Tianjin 510289, China; ggbxxx@139.com; 3 College of Petroleum Engineering, China University of Petroleum (East China), Qingdao 266580, China; chenmingfrac@163.com

**Keywords:** air nanobubble, long-term stability, spectrophotometry, Ångström exponent, characterization methods

## Abstract

The long-term stability of bulk nanobubbles is crucial for their functional applications; however, understanding the evolution of their size distribution remains a significant challenge. While conventional characterization methods, such as Dynamic Light Scattering and Nanoparticle Tracking Analysis, provide size information, they are often sample-intensive and expensive, making them ill-suited for high-throughput or long-term dynamic monitoring of size distribution polydispersity. This research validated UV–Vis spectrophotometry as a simple, powerful tool for tracking these dynamic changes. Air nanobubbles generated via pressure oscillation-hydraulic cavitation were systematically monitored over 30 days using correlative DLS, NTA, and UV–Vis spectroscopy. A distinct two-stage evolution was identified: an initial “purification” phase marked by the dissolution of unstable bubbles, followed by a long-term “maturation” phase governed by Ostwald ripening. The Ångström exponent (*n*), derived from the full extinction spectrum, is a highly sensitive descriptor of this process. The evolution of *n* traced a unique V-shaped trajectory, which resulted in a pronounced hysteresis loop when plotted against the mean diameter from DLS. This hysteresis reveals that systems with identical mean diameters can possess vastly different distribution morphologies, which are inaccessible through traditional sizing methods alone. This research establishes full-spectrum UV–Vis analysis as a robust methodology, enabling rapid and efficient assessment of nanobubble stability and providing a deeper mechanistic understanding of their complex evolution.

## 1. Introduction

Bulk nanobubbles, which are gaseous nuclei dispersed in a liquid with diameters under 1000 nm, have become a subject of intense research [1,2,3]. Their exceptional longevity, lasting for days to months, fundamentally challenges the classical Laplace pressure theory [4]. This anomalous stability, combined with a large specific surface area and a negatively charged gas–liquid interface, enables promising applications in fields ranging from environmental remediation to biomedicine [5,6,7]. Indeed, recent advancements have highlighted their growing importance in diverse areas such as advanced drug delivery [8], catalysis [9], food processing [10], and even complex aerodynamic and heat transfer applications [11,12,13], underscoring the broad impact of understanding their fundamental behaviors. Although various generation techniques are available, such as acoustic, electrochemical, and hydrodynamic methods, the development of efficient, controllable, and cost-effective preparation methods, coupled with a deeper understanding of their stability mechanisms, remains a central issue for advancing this field [14,15,16].

Successful enhanced oil recovery (EOR) applications demand nanobubble populations with high number density, narrow size distributions, and robust long-term stability to ensure their viability during transport deep into reservoirs [17,18,19]. Hydrodynamic generation is a promising technique for field-scale operations owing to its simplicity and low energy footprint. Recent work has indicated the efficacy of various gas nanobubbles (CO_2_, N_2_, and CH_4_) for EOR [20]. Elnaggar et al. experimentally demonstrated that N_2_ nanobubble solutions enhanced oil recovery via spontaneous imbibition in both sandstone and carbonate rocks across a wide range of wettability, temperature, and pressure conditions [21]. Using high-pressure glass micromodels, Taman et al. demonstrated that N_2_ nanobubbles enhance oil sweep efficiency, particularly under oil-wet conditions, by reducing interfacial tension and inducing a wall-slippage effect [22]. Through core flooding experiments and NMR analysis, Zhu et al. determined that CO_2_ nanobubbles yielded higher oil recovery in conglomerate reservoirs than N_2_ nanobubbles by more effectively displacing oil from smaller pores [23]. Cai et al. developed a CO_2_ nanobubble system stabilized by modified nano-SiO_2_ particles that enhanced oil recovery in extra-low-permeability reservoirs by mitigating gas channeling and improving interfacial properties [24]. Despite these advances, the focus has remained on displacement mechanisms rather than the intrinsic stability of the nanobubbles themselves [25,26,27]. Their long-term evolutionary dynamics, particularly under the complex water chemistries found in reservoirs, have not been systematically investigated [28,29,30,31,32]. This deficiency in systematic understanding severely undermines the reliability and scalability of nanobubble EOR technology, hindering its translation from a laboratory concept to a field-ready solution [33,34,35,36,37,38]. This challenge underscores the urgent need for enhanced analytical tools to rapidly assess and predict nanobubble stability [39,40,41,42,43,44].

Accurate characterization of nanobubble stability is fundamental to understanding their behavior and optimizing their performance [45,46,47]. Dynamic Light Scattering (DLS) and Nanoparticle Tracking Analysis (NTA) are the predominant techniques, often used in combination [48,49,50,51,52]. Xu et al. introduced a compression-decompression method to study how super-high dissolved gas concentrations affect bulk nanobubbles. The results revealed that the nanobubble concentration followed a complex evolution, first increasing sharply before gradually decreasing with fluctuation [53]. Ye et al. developed an online dynamic light scattering system for real-time monitoring of nanobubble generation. This technique revealed that bubbles form at a submicron size and then rapidly shrink, reaching a stable size in approximately 35 s [54]. Phan et al. investigated the formation and stability of CO_2_ nanobubbles for potential applications in food processing. Using DLS, they indicated that the nanobubbles had a size of 200–500 nm and were stable for over seven days [55]. Nazari et al. reviewed recent progress in the generation and detection of nanobubbles for mineral flotation. This review identified DLS and NTA as the predominant characterization methods used in laboratory-scale studies [56]. Numerous studies have adopted the combined strategy of DLS and NTA [57,58,59]. Nazari et al. investigated the effect of nanobubbles on the flotation of graphite from lithium-ion batteries. Characterization using DLS revealed that nanobubbles increased the flotation recovery by up to 15% [60]. In coal flotation applications, Jiang et al. investigated the effect of nanobubbles on ultrafine particles at high impeller speeds. NTA revealed that nanobubbles promoted particle aggregation and enhanced flotation even under intense turbulence [61]. Qiao et al. studied the use of nanobubbles for removing detrimental clay coatings from coal. NTA demonstrated that this treatment effectively removed the clay and significantly improved flotation recovery [62]. Focusing on bubble stability, Zhou et al. examined how substances such as detergents and oil affect bubbles from a nanobubble generator. DLS results indicated that detergents increased bubble concentration, while oil appeared to enhance stability [63].

DLS provides the average particle size of a system rapidly; however, its results can be disproportionately skewed by a small number of large particles [64,65]. In contrast, NTA offers high-resolution information on particle size and absolute concentration. However, this requires a specific sample concentration and is time-consuming. As highlighted in relevant reviews, while the DLS-NTA combination has become the standard approach, it has inherent limitations in terms of efficiency and cost when rapid routine screening of multiple preparation conditions or formulations is required. Therefore, exploring a more straightforward, faster, and sensitive new method that can reflect the macroscopic stability of the system has significant practical implications for accelerating the research progress of nanobubble technology.

In this study, air nanobubble suspensions were prepared using a pressure oscillation-hydrodynamic cavitation system. To systematically evaluate their long-term stability, the suspensions were continuously monitored for 30 days. DLS was utilized to track the evolution of the nanobubbles’ mean particle size and zeta potential. NTA was employed at key time points to validate both bubble size and absolute number concentration. Changes in the apparent absorbance of the sample were also simultaneously recorded using UV–Visible (UV–Vis) spectrophotometry.

## 2. Materials and Methods

### 2.1. Nanobubble Preparation

Ultrapure water was prepared using a PINTO PT-RO-20L/H system (Shanghai, China). The resistivity was consistently 18.2 MΩ·cm (25 °C), and it served as the liquid medium for air nanobubble generation. To ensure experimental accuracy and reproducibility, the water had extremely low impurity levels, with a total organic carbon content below 5 ppb, a heavy metal ion concentration not exceeding 0.1 ppb, and a particle count (>0.22 μm) controlled to less than 1 particle/mL.

To minimize potential contamination, all containers and tools that came into contact with the samples were subjected to a rigorous cleaning protocol. First, they were sonicated for 30 min in a laboratory-grade detergent. They were then rinsed thoroughly with tap water and ultrapure water. Subsequently, the equipment was immersed in a 10% (*w*/*v*) sodium hydroxide solution for a minimum of 2 h to remove organic residues. Ultrapure water rinses were repeated until the resistivity of the rinse water matched that of the source water. Finally, vials and stainless needles were autoclaved at 121 °C for 20 min and then dried in a clean environment for later use. Before all solution preparations, ultrapure water was terminally filtered through a 0.22 μm sterile disposable filter to ensure sterility and particle-free conditions. Ambient laboratory air (approx. 78% N_2_ and 21% O_2_) was used as the gas source. To prevent particulate or microbial contamination from affecting nanobubble formation and stability, the air was purified by passing it through a 0.22 μm sterile disposable filter before being introduced into the system.

**Figure 1:**
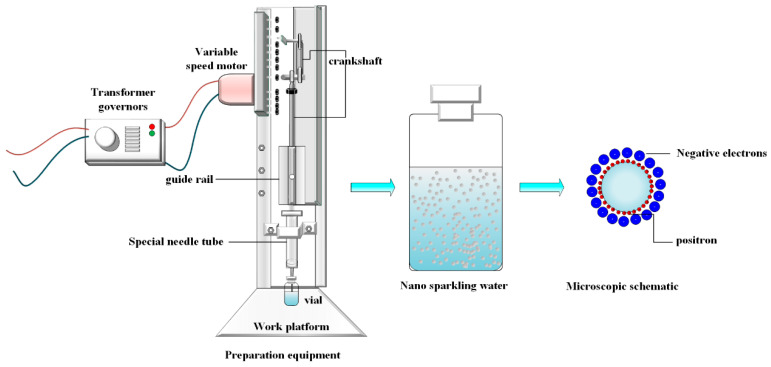
Schematic of the nanobubble generation apparatus based on the Pressure Oscillation-Hydrodynamic Cavitation (POHC) method.

Air nanobubbles were prepared using a custom-designed device based on the Pressure Oscillation-Hydrodynamic Cavitation (POHC) method (Figure 1). The core components include a stepper motor for reciprocating motion, a mechanical arm rigidly connected to a 20 mL syringe piston, a fixed syringe barrel, and a 10 mL sample vial. The syringe needle passed through the vial’s sealed rubber stopper and was immersed below the liquid surface. The connections between the piston and barrel, as well as between the needle and stopper, are airtight, creating a closed pressure oscillation system.

The working principle of the device is as follows. The stepper motor drives the mechanical arm, causing the piston to push and pull periodically within the sealed system. This action induced strong pressure oscillations. During the decompression phase, the abrupt pressure drop causes the dissolved gas in the water to become supersaturated, leading to the nucleation of initial micron-sized bubbles. Subsequently, the forward motion of the piston forces the bubble-laden liquid to pass through the narrow needle at a high velocity. According to Bernoulli’s principle, a dramatic increase in the flow speed generates a local low-pressure zone within the needle, which induces a strong hydrodynamic cavitation effect. The powerful shear forces and shock waves associated with this effect effectively fragment and refine micron-sized bubbles to the nanoscale. This continuous isothermal nucleation-fragmentation cycle ultimately produces a stable nanobubble suspension with a uniform size distribution.

The nanobubbles were prepared following a standard procedure. First, 4 mL of ultrapure water was injected into a 5 mL sterile vial, leaving approximately 1/5 of the total volume as air headspace to serve as the gas source. The vial was then connected to a 20 mL syringe with a pre-set piston stroke via a needle, ensuring that the needle tip was immersed below the liquid surface to form a closed system. The piston was set to a stroke volume of 15 mL, which generated an estimated pressure oscillation within the vial ranging from approximately 50 to 350 kPa. The motor frequency was set to 60 rpm and maintained for 20 min. This frequency was selected following preliminary optimization experiments. Frequencies of 30 and 90 rpm were also tested, but 60 rpm yielded the highest nanobubble concentration and the most uniform size distribution. After preparation, the sample vial was immediately removed from the apparatus. To ensure equilibrium at ambient atmospheric pressure, the headspace was vented briefly before the vial was sealed. The sample was then stored in a dark, temperature-controlled environment at 25 °C for subsequent characterization.

### 2.2. Characterization Methods

#### 2.2.1. UV–Vis Spectrophotometry

The stability of the nanobubble suspension was semi-quantitatively evaluated using a Lichen L9 UV–Visible spectrophotometer (Changsha, China). Before the experiment, the spectrophotometer was warmed for 30 min to ensure a stable light source. Nanobubbles suspended in water act as independent light-scattering centers. This scatters a portion of the incident light away from its original path, which in turn reduces the intensity of the transmitted light reaching the detector. The instrument interprets this reduction in light intensity as “absorbance.” For colloidal systems, such as nanobubbles, which fundamentally do not absorb photons, the displayed “absorbance” is an apparent absorbance dominated by light scattering effects, rather than molecular absorption, as described by the Beer-Lambert law. Theoretically, the apparent absorbance value is directly related to the number density and size of bubbles.

Therefore, by continuously monitoring the change in the absorbance of the sample over time, the aggregation or dissolution of the nanobubble population can be effectively tracked. Ultrapure water was used as a blank reference. A 1 cm path length quartz cuvette was used, and the absorbance of the sample was recorded at a constant temperature of 25 °C over the wavelength range of 200–1100 nm. Continuous monitoring of this apparent absorbance change enables an accurate evaluation of the overall stability of the nanobubble population.

#### 2.2.2. Dynamic Light Scattering Analysis

DLS was used to characterize the hydrodynamic diameter and polydispersity index of the nanobubbles. All measurements were performed using a Nicomp Z3000 nanoparticle size and zeta potential analyzer (Shanghai, China). This technique measures the fluctuations in the scattered light intensity caused by the Brownian motion of the particles in the sample. It then calculates the particle diffusion coefficient and converts it to particle size information using the Stokes-Einstein equation.

The specific measurement parameters were set as follows: The sample was equilibrated at a constant temperature of 23 °C for 3 min before measurement. The scattering angle was set to 90°. The refractive index and viscosity of ultrapure water were set to 1.333 and 0.933 mPa·s, respectively. Considering that the bubble core was composed of gas, the particle refractive index was set to 1.00. To ensure data reliability, each sample was measured independently three times, with each measurement lasting 5 min, and the average value was taken.

A significant characteristic of DLS is that the scattered light intensity is proportional to the sixth power of the particle diameter. This makes the technique extremely sensitive to a small number of large particles or aggregates within the system. To provide a comprehensive and accurate representation of the overall bubble distribution, this study presents both intensity- and number-weighted particle size distribution plots.

#### 2.2.3. Nanoparticle Tracking Analysis

NTA was conducted using a NanoSight NS300 system (Malvern Panalytical Ltd., Malvern, UK) for direct visualization and to obtain high-resolution particle size distributions and absolute number concentrations. The technique operates by tracking the Brownian motion of individual particles and calculating their hydrodynamic diameters from their respective diffusion coefficients using the Stokes-Einstein equation.

A key strength of NTA is its single-particle analysis capability, which provides not only detailed size profiles and absolute concentrations, but also direct visual confirmation of the nanobubbles. The concentration of the as-prepared nanobubble samples fortuitously fell within the instrument’s recommended concentration range of 10^7^–10^9^ particles/mL and size range of 30–1000 nm; therefore, all samples were analyzed directly without dilution.

## 3. Results and Discussion

### 3.1. Characterization of Nanobubble Generation

**Figure 2:**
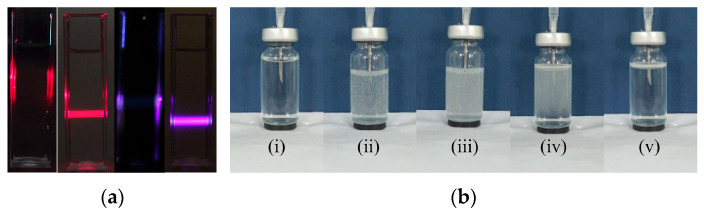
Tyndall effect and macroscopic generation process. (**a**) Verification of nanobubble generation via the Tyndall effect. Ultrapure water (left, control) and the nanobubble suspension (right) were illuminated by 650 nm (red) and 405 nm (purple) lasers. (**b**) Evolution of macroscopic phenomena during nanobubble preparation using the POHC method. (i) The initial state, showing the clear, ultrapure water in the vial before the process begins; (ii) the solution begins to turn turbid as the microbubbles and nanobubbles are nucleated under pressure oscillations; (iii) the solution reaches a saturated, milky-white state, indicating a high concentration of bubbles generated by the continuous process; (iv) the milky turbidity of the solution begins to decrease, microbubbles in the solution gradually disappear during the depressurization process; (v) the solution exhibits a uniform, stable, translucent ‘misty’ appearance, the microbubbles have fully disappeared when depressurization is complete.

Following the treatment of ultrapure water with the POHC method, nanobubbles were generated using multiple characterization techniques. The Tyndall effect provides the most intuitive evidence (Figure 2a). When a 405 nm violet laser beam and a 650 nm red laser beam were passed through untreated ultrapure water, no discernible light path was observed. However, a precise bright rectangular light beam was distinctly visible when the same conditions were applied to the POHC-treated sample. This observation preliminarily demonstrates the presence of colloid-sized particles capable of significant light scattering.

As a control, untreated ultrapure water showed a minimal and irregular DLS signal, which ruled out the possibility of significant scattering contributions from background impurities. Therefore, the intense light scattering signal observed after the POHC treatment was primarily attributed to the newly formed air nanobubbles.

During the nanobubble preparation using the POHC device, a series of systematic physical changes were observed. In the initial cycles (approximately the first five strokes), the solution gradually transitioned to a milky white, turbid state. This phenomenon became more pronounced in each subsequent cycle (Figure 2b). As the cycles continued, a rapid transition from a clear, transparent solution to a milky-white, turbid state was observed during the downward pressurizing stroke of the piston. This indicates the instantaneous nucleation of a large number of micro- and nanosized bubbles under high pressure. Conversely, during the upward, depressurizing stroke, the system pressure decreased, causing some larger, unstable microbubbles to aggregate. These aggregates then rapidly ascended and ruptured owing to buoyancy, temporarily reducing the milky-white appearance of the solution.

After the device was stopped at a predetermined time, the large, visible bubbles disappeared completely within a short period. The entire solution then took on a stable, uniform “misty” appearance. This misty state persisted for several days, providing visual confirmation that a large number of tiny, slow-moving nanobubbles had formed a stable colloidal suspension in water.

**Figure 3:**
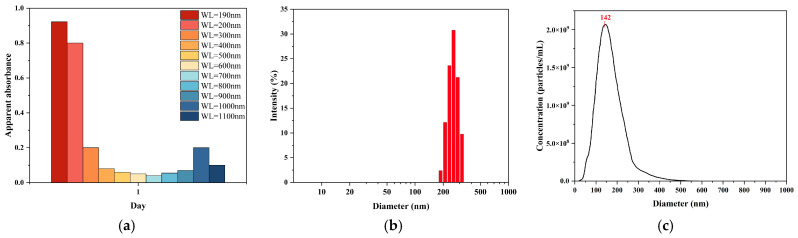
Initial characterization of the as-prepared nanobubble suspension on Day 1. (**a**) Apparent absorbance spectrum. (**b**) Intensity-weighted particle size distribution by DLS. (**c**) Particle size and concentration distribution by NTA.

UV–Vis spectrophotometry was employed to provide further evidence of nanobubble formation and evaluate its potential as a rapid assessment tool (Figure 3a). The POHC-treated sample produced a spectrum characteristic of colloidal dispersion, indicating the presence of nanobubbles. While the ultrapure water control showed a flat baseline with near-zero absorbance from 200 to 1100 nm, the nanobubble sample exhibited a unique spectral shape: a maximum apparent absorbance at 200 nm (A = 0.467), a steep decline to a stable plateau (A ≈ 0.03–0.04) across the visible range (400–700 nm), and a subsequent rise in the near-infrared region. This profile is a hallmark of light scattering by nanoscale particles rather than molecular absorption, confirming the presence of a high concentration of nanobubbles.

The results from the DLS and NTA measurements strongly supported this conclusion. To further quantify the particle size, the sample was subjected to DLS analysis. Based on the intensity-weighted size distribution (Figure 3b), the nanobubble system exhibited a well-defined and relatively concentrated single-peak distribution. The primary intensity peak was located between 200 and 300 nm, with a calculated Z-average (intensity-weighted mean diameter) of 241.8 nm. These results indicate the successful preparation of a nanobubble suspension with good size uniformity via the POHC method.

NTA revealed a particle concentration of 2.79 × 10^9^ particles/mL with a mean size of 165.5 nm (Figure 3c). This particle concentration accounts for the observed light scattering. Furthermore, DLS measurements indicated the system’s polydispersity, yielding a number-weighted average diameter of 51.7 nm and an intensity-weighted average diameter of 312.9 nm. This disparity indicates the presence of a large population of smaller nanobubbles (approximately 50 nm) and a smaller population of larger ones. The latter dominates the intensity-weighted average owing to the sixth-power dependence of the scattering intensity on the particle diameter. This highly polydisperse colloidal system produces the characteristic extinction profile observed in the UV–Vis spectrum. Therefore, UV–Vis spectrophotometry is a simple and rapid method for qualitatively assessing nanobubble generation.

While direct methods for nanobubble verification exist, such as rarefaction wave experiments, they require specialized instrumentation that was not employed in this study. Accordingly, the gaseous nature of the particles was established based on three key lines of indirect evidence: (1) the generation principle, where particles nucleate from dissolved gas in a clean, closed system, precludes solid contaminants; (2) the system exhibits a characteristic Tyndall effect and Ostwald ripening dynamics, wherein the particle number decreases as the mean size increases; (3) the macroscopic evolution from a milky to a stable misty suspension is consistent with a colloidal gas dispersion. This body of complementary evidence provides strong support for the conclusion that the observed particles were nanobubbles.

### 3.2. Long-Term Stability and Evolution Mechanism of Nanobubbles

**Figure 4:**
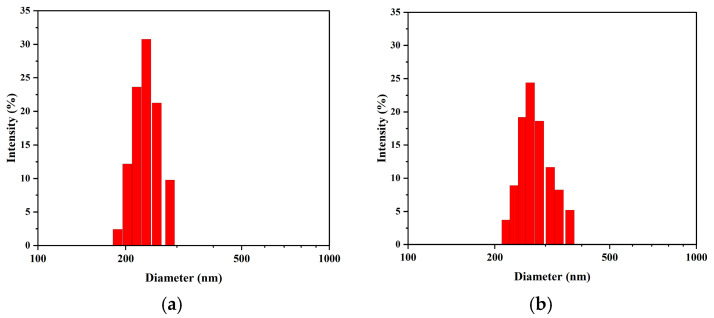

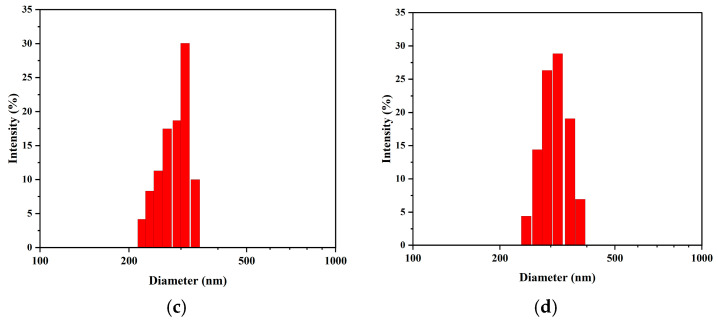
Representative intensity-weighted particle size distributions measured by DLS at key time points: (**a**) Day 1, (**b**) Day 10, (**c**) Day 20, and (**d**) Day 30.

**Figure 5:**
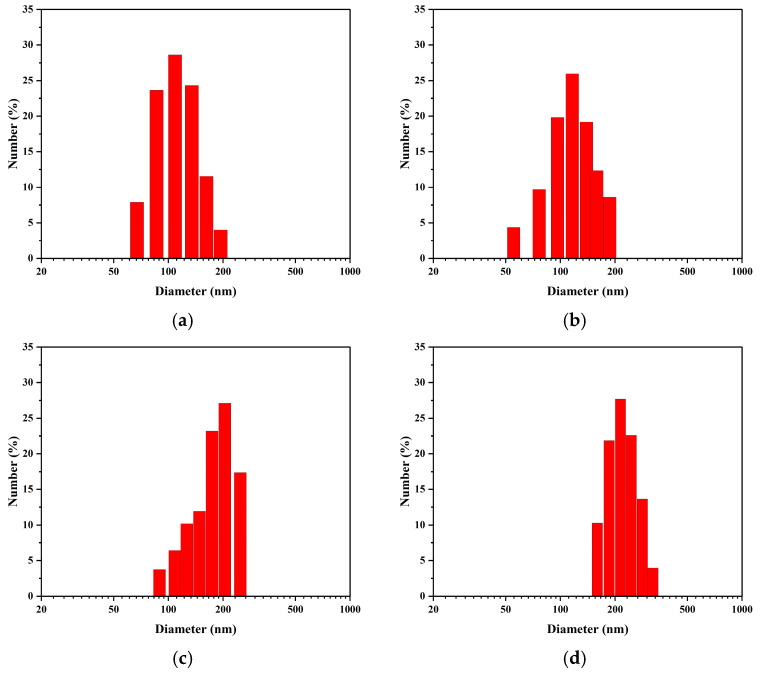
Representative number-weighted particle size distributions measured by DLS at key time points: (**a**) Day 1, (**b**) Day 10, (**c**) Day 20, and (**d**) Day 30.

To systematically evaluate the long-term stability of the nanobubbles prepared by the POHC method, 30-day continuous DLS monitoring was performed on the same sample. As shown in Figure 4, Figure 5, Figures S1 and S2, the evolution of the nanobubble system exhibited distinct stages and maintained stability after the initial adjustment period. The number-weighted mean diameter underwent an initial “purification phase” during the first week, decreasing from 101.4 nm to a minimum of approximately 82 nm. This initial decrease was attributed to the rapid dissolution of a small population of larger, thermodynamically unstable bubbles. Subsequently, the system entered a slow “ripening phase,” during which the number-weighted mean diameter gradually increased to approximately 103 nm over the following three weeks.

In contrast, the intensity-weighted mean diameter increased continuously over the 30-day period. It increased from an initial value of approximately 242 nm to a final value of 345 nm. This divergence between the number- and intensity-weighted trends is characteristic of an Ostwald ripening process (Figure 4b,c). After the initial purification phase, a core population of stable nanobubbles (80–100 nm) remained. These numerous bubbles then began to grow slowly by incorporating gas from smaller, dissolving bubbles, leading to a gradual increase in their overall size. This growth is particularly evident in intensity-weighted data for a specific reason. According to scattering theory, DLS signal intensity is proportional to the sixth power of the particle diameter. Consequently, the growth of even a small number of larger bubbles disproportionately increases the intensity-weighted average. Thus, the DLS data revealed a two-stage evolutionary pathway: a rapid purification phase followed by a slow-ripening phase. The stability of the core population indicates that the nanobubble suspension possesses favorable long-term storage properties.

To further investigate the long-term evolution mechanism and to complement the DLS measurements, the sample size and absolute number concentration were tracked using an NTA instrument at key time points (days 1, 10, 20, and 30). NTA results revealed the dynamic evolution of the system. Over the 30-day monitoring period, the number-weighted mean diameter remained relatively stable, increasing from an initial value of 165.5 nm to 174.1 nm. However, during the same period, the number concentration of nanobubbles continuously decreased from 2.79 × 10^9^ particles/mL to 1.08 × 10^9^ particles/mL on Day 30.

These two divergent trends are consistent with the Ostwald Ripening mechanism. The relative constancy of the mean diameter indicates the intrinsic stability of the core nanobubble population, which constitutes the majority of the particles. Conversely, the continuous decrease in concentration directly reflects the ongoing dissolution of the smallest bubbles, which have the highest internal pressure. The gas molecules from these dissolving bubbles diffuse and are absorbed by slightly larger bubbles. Because only the smallest bubbles dissolve while the main population’s size is unchanged, the impact on the number-weighted average diameter is negligible. Thus, NTA measurements quantitatively supported the long-term stability of the POHC-prepared nanobubbles. Furthermore, these results provide a direct mechanistic explanation for the slow increase observed in the DLS intensity-weighted average diameter.

**Figure 6:**
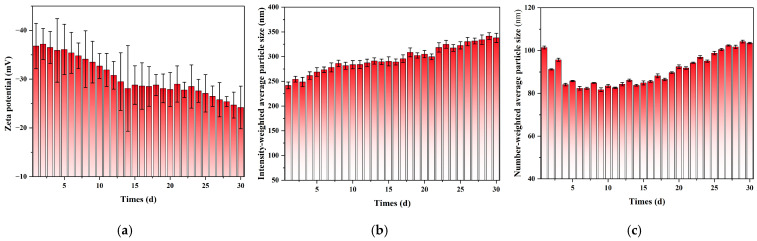
Long-term stability of nanobubbles monitored by DLS over 30 days. (**a**) Evolution of Zeta potential. (**b**) Evolution of the intensity-weighted average particle size (Z-Average). (**c**) Evolution of the number-weighted average particle size.

To investigate the electrostatic mechanism behind the long-term stability of nanobubbles, the system’s zeta potential was monitored continuously for 30 days. As shown in Figure 6a, the zeta potential exhibited a systematic change over time. In the initial preparation stage, the nanobubbles showed a highly negative potential of approximately −37 mV. This is primarily attributed to the preferential adsorption of hydroxyl ions OH^−^ at the gas–liquid interface, which forms a stable electrical double layer on the bubble surface. This strong electrostatic repulsion is the fundamental reason for preventing rapid bubble coalescence in the initial phase and ensuring the macroscopic stability of the system.

Over the 30-day monitoring period, the absolute value of the Zeta potential showed a gradual but continuous decreasing trend, reaching approximately −24 mV by Day 30. Although this value is at the threshold of what is typically considered sufficient for long-term colloidal stability (i.e., less negative than −25 mV), the substantial surface charge was still adequate to prevent rapid, large-scale coalescence throughout the observation period. This explains why the system’s evolution was dominated by the slow, diffusion-driven mechanism of Ostwald Ripening rather than a catastrophic aggregation event. However, the continuous downward trend suggests that over timescales longer than 30 days, this weakening electrostatic barrier may allow coalescence to play an increasingly significant role in the ultimate destabilization of the system.

From the perspective of colloidal stability, although the absolute value of the zeta potential decreased, it consistently remained at a level sufficient to provide effective electrostatic repulsion throughout the monitoring period (generally >−25 mV). This perfectly explains the phenomena observed in the DLS and NTA results; the system did not undergo catastrophic, coalescence-dominated destabilization, but rather a slow, diffusion-driven Ostwald Ripening process. Therefore, the persistent and sufficiently strong negative charge on the surface of nanobubbles prepared using the POHC method is the core electrostatic basis that enables them to resist coalescence and achieve macroscopic stability for weeks or even months.

**Figure 7:**
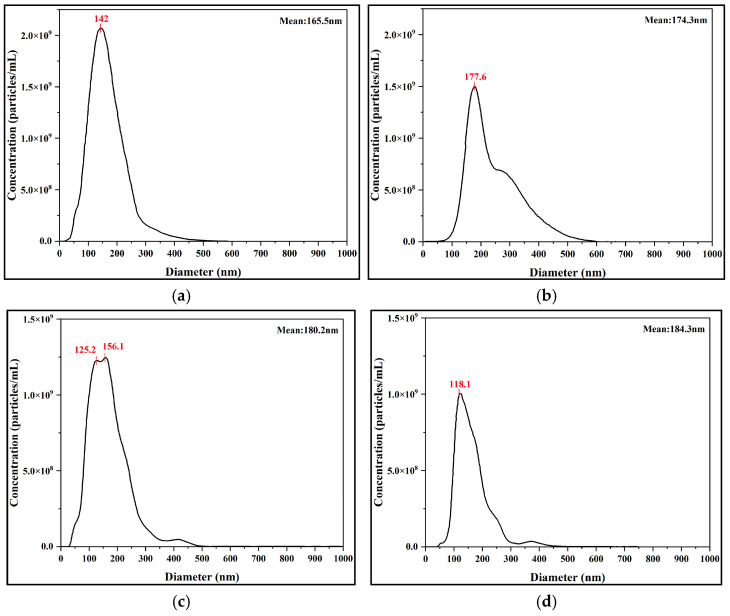
Evolution of the nanobubble size and concentration distribution at key time points measured by NTA. (**a**) Day 1. (**b**) Day 10. (**c**) Day 20. (**d**) Day 30.

Furthermore, analysis of the NTA data provides a complementary and consistent view of particle size evolution (Figure 7). The number-weighted mean diameters were determined to be 165.5 nm (Day 1), 174.3 nm (Day 10), 18.2 nm (Day 20), and 184.3 nm (Day 30). This revealed a slow but steady increase in the average nanobubble size over the 30-day period. This trend is in excellent agreement with the increasing intensity-weighted size observed via DLS (Figure 6b). Therefore, both independent measurement techniques consistently support an Ostwald ripening mechanism, where larger bubbles grow as the overall number concentration decreases.

### 3.3. Spectrophotometric Monitoring and Correlation Analysis

**Figure 8:**
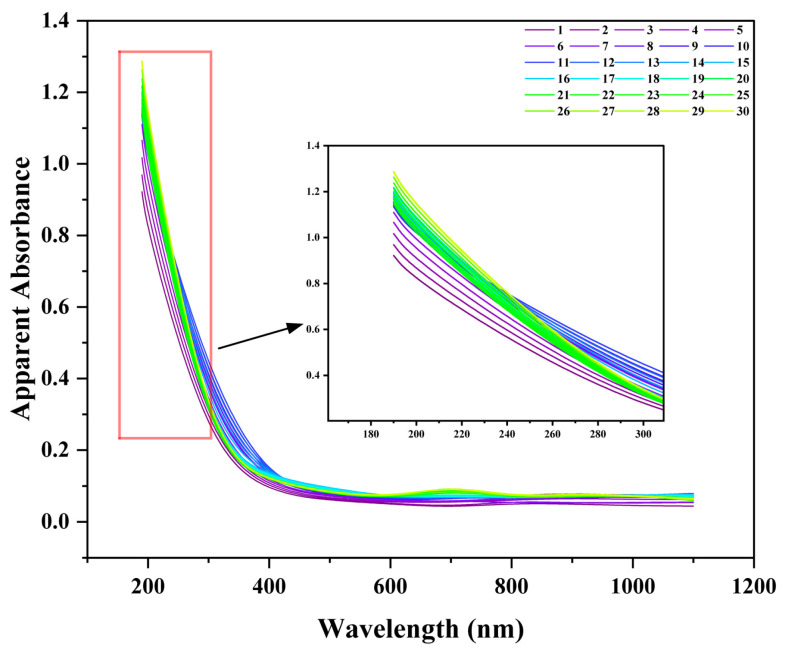
Evolution of the apparent absorbance spectra of the nanobubble suspension over 30 days. The inset shows a magnified view of the 180–300 nm region.

To establish a rapid method for assessing nanobubble stability, 30-day continuous UV–Visible spectrophotometry monitoring was performed. The absorbance results were compared with the intensity-weighted mean diameter (Z-average) obtained from DLS. The daily absorbance spectra exhibited a typical scattering profile, with maximal absorbance in the deep UV region (e.g., 190 nm), which decayed with increasing wavelength (Figure 8). This spectral shape is consistent with the Rayleigh or Mie scattering theory for nanosized colloids, indicating the presence of nanobubbles.

The evolution of the apparent absorbance at 190 nm and the Z-average were tracked over time. Both metrics showed continuous and parallel increases throughout the 30-day study period. Specifically, the absorbance increased from 0.922 to 1.181, while the Z-average grew from 241.8 nm to 345.3 nm. A strong positive correlation was observed between these two independent measurements (Pearson’s R^2^ > 0.95), suggesting that both techniques monitor the same physical process: Ostwald ripening. The increase in the DLS Z-average reflects the growth of larger bubbles at the expense of smaller ones. Concurrently, the increase in the apparent absorbance is a macroscopic response to this size evolution, as the total light scattering cross-section of the system is enhanced. Therefore, the apparent absorbance measured using UV–Vis spectrophotometry can sensitively reflect the long-term evolution of a nanobubble system driven by Ostwald ripening. This validates the technique as a rapid and simple tool for the semi-quantitative assessment of nanobubble stability.

### 3.4. Validation of UV–Vis Spectrophotometry as a Rapid Assessment Tool

While the absorbance at a single wavelength reflects the overall evolution of the system, the entire spectral morphology can provide more quantitative information. For scattering systems of nanoparticles, the apparent absorbance (A) typically follows a power-law relationship with the wavelength (λ), expressed as A(λ) ∝ λ^−*n*^. The Ångström exponent *n* is a dimensionless parameter related to the morphology of the particle size distribution.

**Figure 9:**
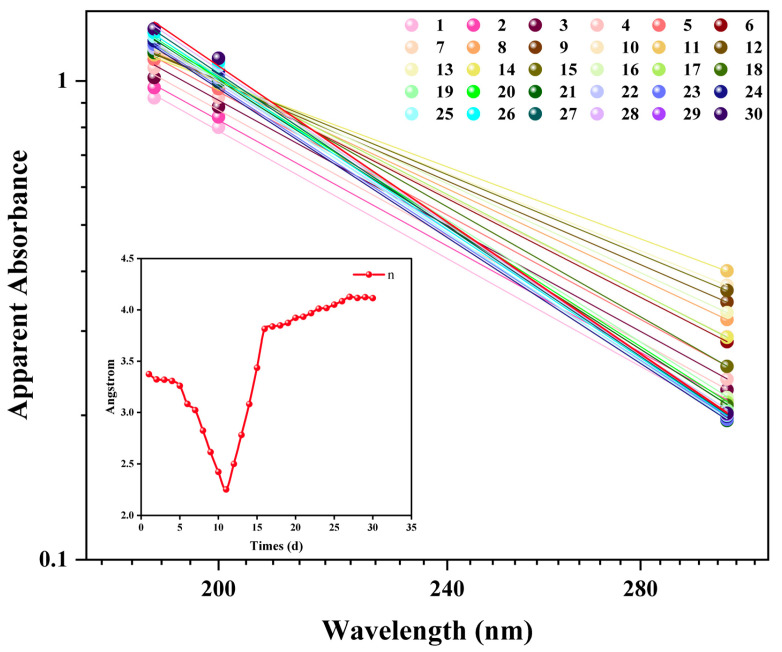
Derivation and evolution of Ångström exponent (*n*) from apparent absorbance spectra. The main panel shows the log-log plot for linear fitting, and the inset shows the calculated *n* value as a function of time.

The evolution of *n* over 30 days exhibited a distinct, non-monotonic, two-phase characteristic (Figure 9). In the first phase (Days 1–11), *n* decreased from 3.37 to a minimum of 2.25. This trend is consistent with the initial evolution observed by DLS, which showed a simultaneous “purification” (number-weighted diameter decreased) and “ripening” (intensity-weighted diameter increased) process. The decrease in *n* is a macroscopic response to the increasing average scattering size of the system during this phase.

In the second phase (days 11–30), a notable phenomenon was observed. Although both DLS-measured mean diameters continued to increase, the Ångström exponent reversed its trend and increased from 2.25 to approximately 4.1. This divergence indicates that *n* is sensitive not only to the mean particle size but also to the width and morphology of the size distribution. It is hypothesized that the later stages of ripening are accompanied by narrowing of the particle size distribution. Consistent with this hypothesis, the Polydispersity Index (PDI) was observed to decrease from 0.28 to 0.21 between days 11 and 30, indicating a narrowing of the size distribution during the ripening phase. This PDI-supported narrowing of the size distribution is characteristic of late-stage Ostwald ripening. Such a morphological change would alter the wavelength-dependent light scattering, leading to an observed increase in the Ångström exponent. Although this interpretation is experimentally consistent, a comprehensive Mie scattering model is required for full validation, which is an important direction for future research.

**Figure 10:**
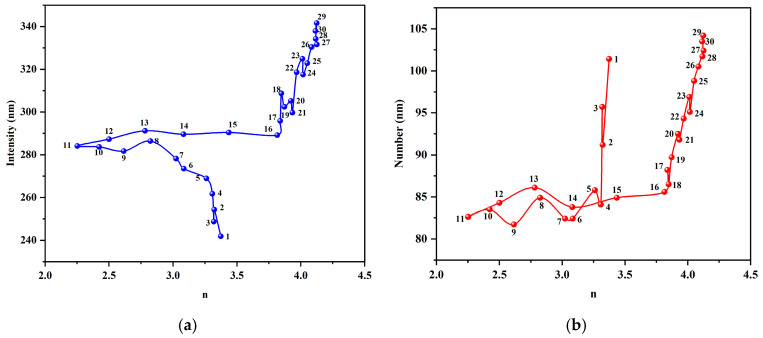
Correlation analysis between the Ångström exponent (*n*) and DLS-measured average particle sizes. (**a**) shows the relationship between *n* and intensity-weighted sizes for 30 days. (**b**) shows the relationship between *n* and number-weighted sizes for 30 days.

To validate this inference, we plotted the relationship between the Ångström exponent *n* and the two DLS-measured mean diameters (Figure 10). Throughout the evolution, the relationship between the value and both the intensity-weighted (Z-average) and number-weighted diameters was not a simple linear relationship. Instead, it formed a unique “hysteresis loop” trajectory. This demonstrates that the Ångström exponent, *n*, is capable of capturing dynamic information about the evolution of the particle size distribution morphology that the simple DLS mean values cannot fully reflect.

**Figure 11:**
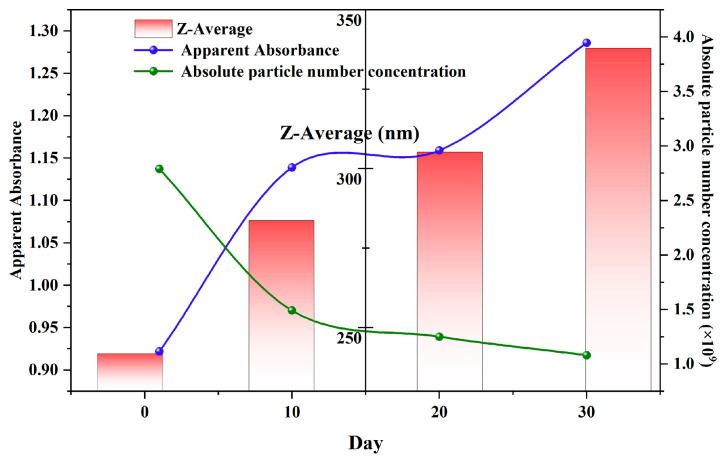
Multi method evolution trajectory of nanobubbles within 30 days.

To provide a more holistic and accessible view of the long-term stability mechanism, the key evolutionary parameters obtained from UV–Vis, DLS, and NTA were integrated into a single timeline (Figure 11). This composite view reveals a clear interplay between the different physical properties of the nanobubble system. A strong positive correlation was immediately evident between the intensity-weighted mean diameter (Z-average) from DLS and the apparent absorbance at 190 nm from UV–Vis spectroscopy. Both metrics exhibit a continuous increase over the 30-day period, confirming that they monitor the same macroscopic consequence of the Ostwald ripening process—the growth of larger bubbles.

Concurrently, NTA data provide a direct mechanistic explanation for this growth. The absolute number concentration of nanobubbles shows a steady decline, decreasing by over 60% from 2.79 × 10^9^ particles/mL to 1.08 × 10^9^ particles/mL. This reduction in particle count, occurring while the average size increases, is the definitive signature of Ostwald ripening, where smaller, thermodynamically less stable bubbles dissolve, providing gas that feeds the growth of their larger counterparts. The integrated analysis therefore validates that the simple, rapid UV–Vis measurement is a reliable proxy for tracking the complex, diffusion-driven evolution of the nanobubble population, which is corroborated by both the size and concentration data from DLS and NTA.

In summary, this study not only validates the use of spectrophotometry for tracking the macroscopic evolution of nanobubbles, but more importantly, demonstrates through the analysis of the Ångström exponent that it is a powerful tool capable of sensitively reflecting dynamic changes in the morphology of the size distribution. Compared to DLS and NTA, it offers a unique and complementary analytical dimension, being rapid, simple, and low-cost. This makes it an ideal, rapid, and cost-effective screening method for assessing and understanding the complex stability mechanisms of nanobubbles.

## 4. Conclusions

This study successfully prepared high-concentration (initial value: 2.79 × 10^9^ particles/mL) and highly stable air nanobubbles using a Pressure Oscillation-Hydrodynamic Cavitation (POHC) method. A 30-day systematic monitoring revealed a complex two-stage evolution mechanism: an initial “purification” phase where the number-weighted mean diameter decreased to a minimum of ~82 nm, followed by a long-term “ripening” phase dominated by Ostwald ripening, during which the intensity-weighted mean diameter (Z-Average) increased from 241.8 nm to 345.3 nm. Throughout this process, a sustained zeta potential above −24 mV provided the necessary electrostatic repulsion to prevent coalescence.

The core contribution of this study is the validation of UV–Vis spectrophotometry as a rapid and information-rich tool for assessing nanobubble stability. We established a strong positive correlation (R^2^ > 0.95) between the apparent absorbance at 190 nm and the Z-average. More critically, we demonstrated that the Ångström exponent (*n*), derived from the full spectral morphology, reveals dynamic information inaccessible to single-size parameters. The *n* value exhibited a unique V-shaped reversal trend (decreasing from 3.37 to 2.25 before rising to ~4.1) and formed a distinct hysteresis loop when plotted against DLS data, proving its exceptional sensitivity to subtle changes in the size distribution morphology.

It is important to acknowledge that this study was conducted using an idealized system of air nanobubbles in ultrapure water. This approach is essential for isolating the fundamental stability mechanisms and validating the spectrophotometric methodology without confounding variables. However, for practical applications, such as in enhanced oil recovery (EOR), where reservoir brines are complex, the effects of environmental factors are critical. We anticipate that increased salinity would compress the electrical double layer, potentially reducing the magnitude of the zeta potential and lowering the barrier to coalescence. The pH of the medium directly influences the surface charge by altering the relative concentrations of H^+^ and OH^−^ ions. Furthermore, the presence of dissolved organic matter could have a dual effect: some molecules might adsorb to the bubble surface and act as surfactants, enhancing stability, whereas others might interact with and neutralize the surface charge. Therefore, future work should focus on systematically investigating the influence of these variables on nanobubble stability and determining the operational window for UV–Vis monitoring in more complex aqueous environments. The ability to efficiently track the complex evolution of nanobubble populations is equally critical for ensuring quality and performance in other advanced applications, including the development of stable contrast agents in biomedicine, optimization of flotation processes in water treatment, and fabrication of novel materials in materials processing. Therefore, this rapid assessment method represents a key enabling tool for accelerating innovation across the diverse fields of nanobubble technology.

## Data Availability

The data of this article are available from the corresponding author upon reasonable request.

## Supplementary Materials

The following supporting information can be downloaded at: https://www.mdpi.com/article/10.3390/nano15211613/s1, Figure S1: Evolution of the intensity-weighted particle size distribution measured by DLS over 30 days; Figure S2.: Evolution of the number-weighted particle size distribution measured by DLS over 30 days.

## Abbreviations

The following abbreviations are used in this manuscript:

POHC	Pressure oscillation-hydraulic cavitation
NTA	Nanoparticle tracking analysis
DLS	Dynamic light scattering
