# Supplementary material for: Long-Term Stability of Nanobubbles Generated via Pressure Oscillation-Hydrodynamic Cavitation: A Rapid Assessment by UV–Vis Spectrophotometry
Source: Nanomaterials (Basel). 2025 Oct 23;15(21):1613. doi: 10.3390/nano15211613 (PMC12608798; doi:10.3390/nano15211613)
Supplement: Supplementary file 1 [file nanomaterials-15-01613-s001.zip › nanomaterials-3887257-supplementary.pdf]

nanomaterials

Article

Long-Term Stability of Nanobubbles Generated via Pressure Oscillation-Hydrodynamic Cavitation: A Rapid Assessment by UV–Vis Spectrophotometry

Lei Huang, Jiaqi Dong, Ming Chen, Lei Li and Ruichao Zhang*

[1] College of Intelligent Manufacturing and Control Engineering, Shandong Institute of Petroleum and Chemical Technology, Dongying 257061, China; 2015016@sdipct.edu.cn (L.H.); 2016003@sdipct.edu.cn (L.L.)
[2] PipeChina Engineering Technology Innovation Co., Ltd., Tianjin 510289, China; ggbxxx@139.com
[3] College of Petroleum Engineering, China University of Petroleum (East China), Qingdao 266580, China; chenmingfrac@163.com
* Correspondence: zrcupc@sdipct.edu.cn

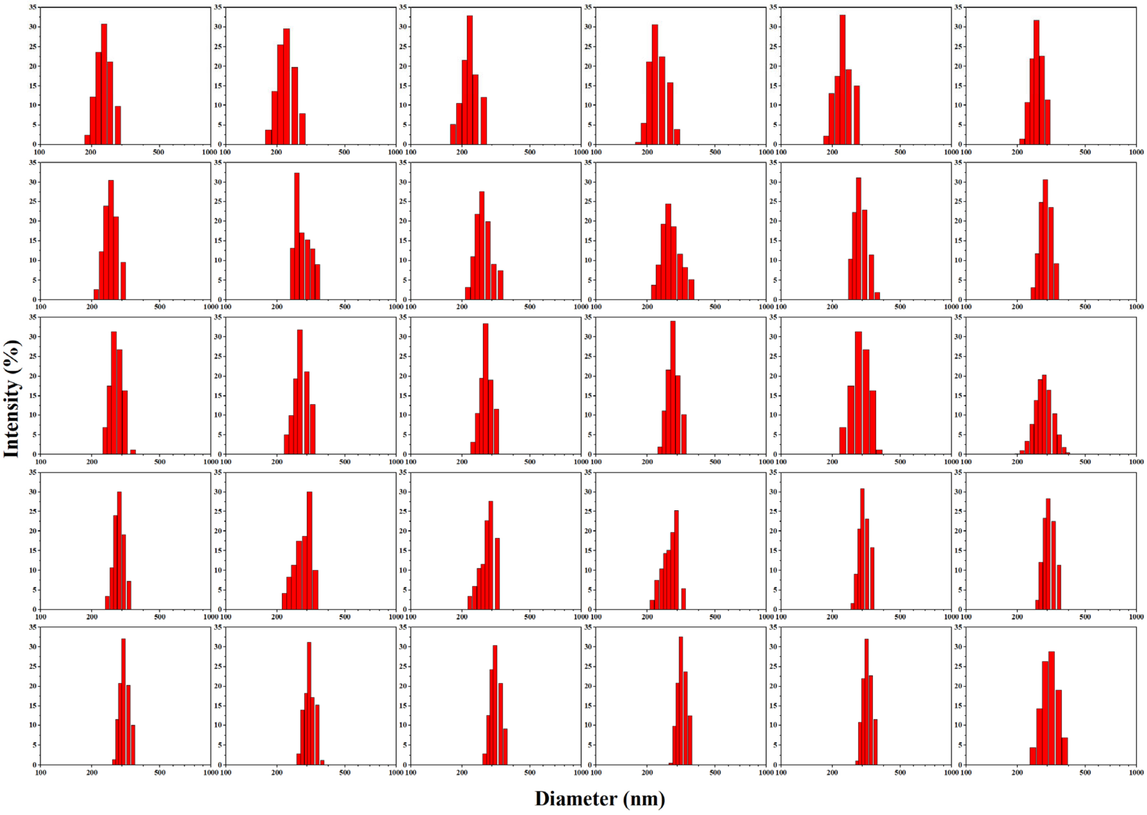

Figure S1. Evolution of the intensity-weighted particle size distribution measured by DLS over 30 days

 https://doi.org/10.3390/nano15211613

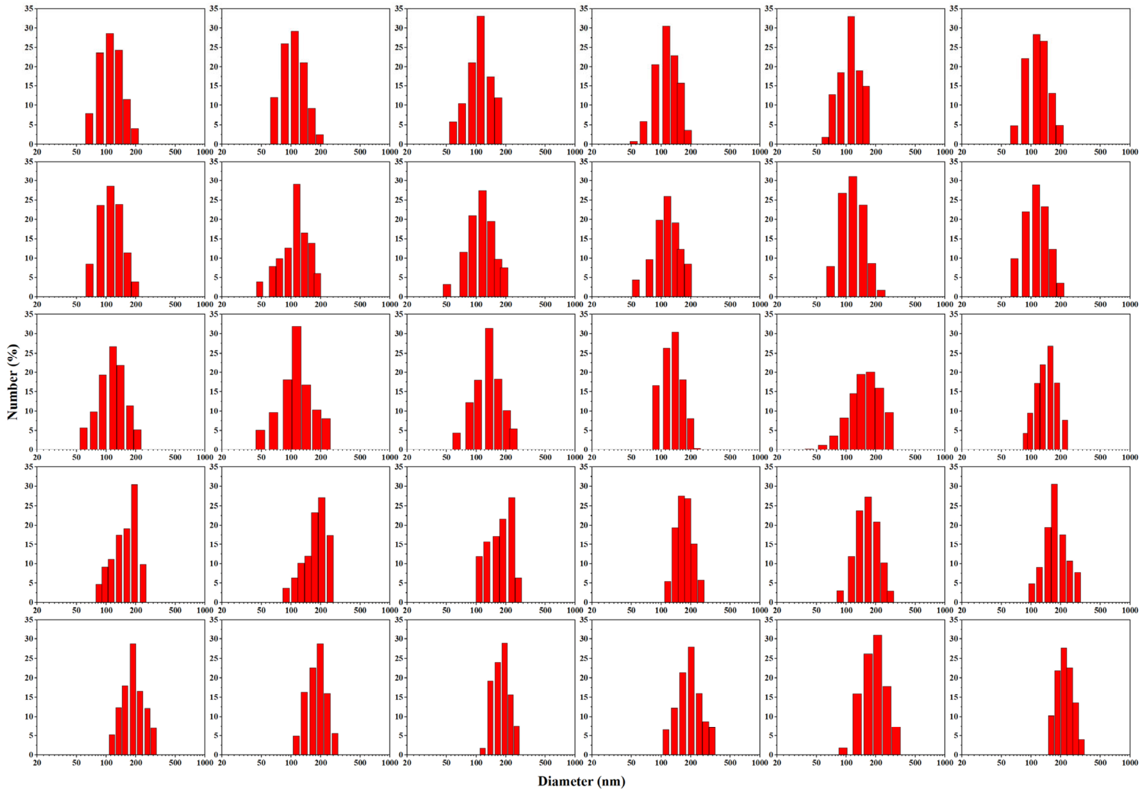

Figure S2. Evolution of the number-weighted particle size distribution measured by DLS over 30 days
